# Inherent Dynamics of the Acid-Sensing Ion Channel 1 Correlates with the Gating Mechanism

**DOI:** 10.1371/journal.pbio.1000151

**Published:** 2009-07-14

**Authors:** Huaiyu Yang, Ye Yu, Wei-Guang Li, Fang Yu, Hui Cao, Tian-Le Xu, Hualiang Jiang

**Affiliations:** 1 Drug Discovery and Design Center, State Key Laboratory of Drug Research, Shanghai Institute of Materia Medica, Chinese Academy of Sciences, Shanghai, China; 2 Institute of Neuroscience and State Key Laboratory of Neuroscience, Shanghai Institutes for Biological Sciences, Chinese Academy of Sciences, Shanghai, China; 3 School of Pharmacy, East China University of Science and Technology, Shanghai, China; University of Texas at Austin, United States of America

## Abstract

The acid-sensing ion channel 1 (ASIC1) is a key receptor for extracellular protons. Although numerous structural and functional studies have been performed on this channel, the structural dynamics underlying the gating mechanism remains unknown. We used normal mode analysis, mutagenesis, and electrophysiological methods to explore the relationship between the inherent dynamics of ASIC1 and its gating mechanism. Here we show that a series of collective motions among the domains and subdomains of ASIC1 correlate with its acid-sensing function. The normal mode analysis result reveals that the intrinsic rotation of the extracellular domain and the collective motions between the thumb and finger induced by proton binding drive the receptor to experience a deformation from the extracellular domain to the transmembrane domain, triggering the channel pore to undergo “twist-to-open” motions. The movements in the transmembrane domain indicate that the likely position of the channel gate is around Leu440. These motion modes are compatible with a wide body of our complementary mutations and electrophysiological data. This study provides the dynamic fundamentals of ASIC1 gating.

## Abstract 2

A combination of computational and experimental approaches reveals the dynamics of ASIC1 gating, involving a deformation of the channel that triggers “twist-to-open” motions of the channel pore.

## Author Summary

The acid-sensing ion channels (ASICs) are key receptors for extracellular protons and are becoming increasingly important drug targets. However, their gating mechanism is still not fully understood. The crystallographic structure of the ASIC1 protein provides a clue, but the dynamics of the channel remains to be elucidated. Using computational biology, site-directed mutagenesis, and electrophysiological recordings, we investigated the dynamics of ASIC1 gating. Through “normal mode analysis,” we detected a series of collective motions between the beta turn and transmembrane domain, and between the thumb and finger domains, suggesting a deformation pathway related to channel gating. The intrinsic rotation of the extracellular domain and the collective motions between the thumb and finger domains that are induced by proton binding serve to deform the channel from the extracellular to the transmembrane domain, triggering a “twist-to-open” motion of the channel pore. The relationship between the dynamics and the gating mechanism was experimentally confirmed by a series of complementary mutations in ASIC1 and electrophysiological measurements. Our study also indicated that the likely position of the channel gate is around Leu440 within the ASIC1 protein. We propose a clear model correlating the structural dynamics of ASIC1 and its gating mechanism.

## Introduction

Extracellular acidosis has profound effects on neuronal function, and acid-sensing ion channels (ASICs) are the key receptors for extracellular protons [1],[2]. ASICs are members of the degenerin/epithelial channel family, which transport Na^+^ through the cell membrane [1],[3], and serve as a paradigm for all proton-gated channels. Six ASIC isoforms, 1a, 1b, 2a, 2b, 3, and 4, have been identified, among which 1a, 2a, and 2b are expressed in the central nervous system (CNS) [2],[4]. In the CNS, ASICs are tightly connected to synaptic plasticity as well as learning and memory in the brain [5],[6]. In addition, it has been demonstrated that activation or sensitization of Ca^2+^-permeable ASIC1a channels are responsible for acidosis-mediated ischaemic brain injury [7],[8] and neuroinflammatory damage [2],[9]. ASICs are therefore becoming increasingly important drug targets [2],[10].

While studies have led to the characterization of ASICs and have furthered the role that they play in neurological diseases, one of the remaining challenges is to fully elucidate their gating mechanisms, which are critical for understanding their biological functions and for developing effective therapeutics [2]. These studies are challenged by the complicated process of ASIC gating: it is proton concentration-dependent, can be blocked by amiloride, and its sodium selectivity and variations of desensitization differ from subtype to subtype [2]. In addition, investigations of the ASIC1 gating mechanism have advanced slowly because of the lack of detailed structural information at atomic resolution. The recent low-pH crystal structure of the chicken ASIC1 (cASIC1) at 1.9 Å resolution has revealed the overall organization of the ASIC1, which provides a framework for probing the mechanism underlying the gating of ASICs [11].

**Figure 1:**
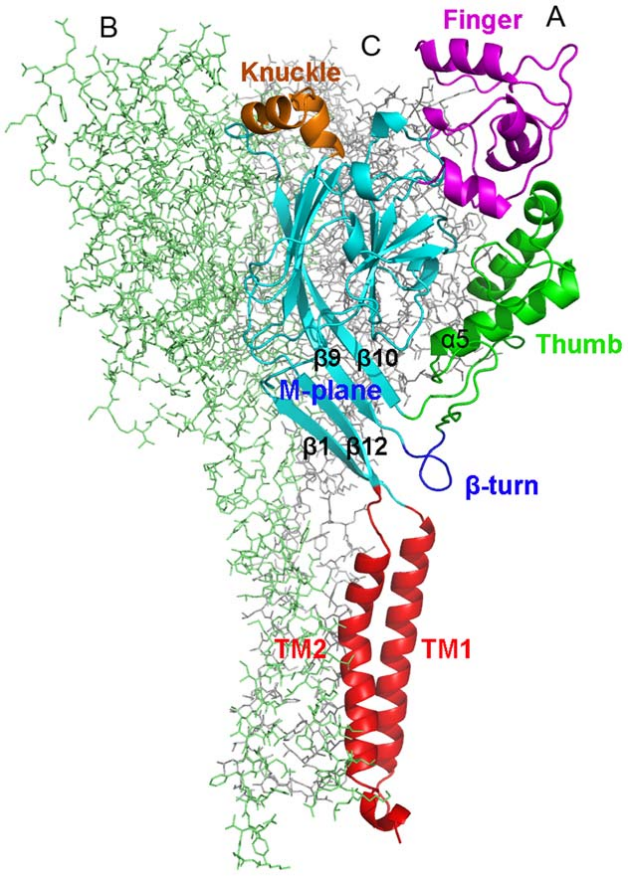
Stereoview of the 3-D structure of cASIC1. The structure model constructed on the basis of the X-ray crystal structure of cASIC1 [11] is shown parallel to the membrane layer. Domain organization of subunit A is indicated in colorful cartoon view, which displays the overall arrangement of different subdomains and important regions. Subunits B and C are displayed in green and gray lines, respectively. This figure and following Figures 2–6 are made with PyMol (http://www.pymol.org).

The crystal structure of cASIC1 revealed that receptors in the superfamily are homo- or heterotrimers [11]. Structurally, the ASIC1 has three subunits with a stoichiometry α_3_, forming a chalice-like architecture. Each subunit is composed of two domains, a large extracellular (EC) domain, and a transmembrane (TM) domain. The EC domain resembles a clenched hand, which can be further divided into finger, thumb, palm, knuckle, and β-turn subdomains. The TM domain comprises two transmembrane helices, TM1 and TM2, in a “forearm” arrangement (Figure 1). This structure has provided insight into the architecture of ASICs and raises intriguing questions about its gating mechanism. For example, what is the function of the large EC domain? Where is the gate located? How is proton concentration sensed by the channel and how does this process trigger opening and closing of the channel? In particular, the intrinsic dynamics of the receptor underlying the gating mechanism is still unclear.

Computational simulation has been a promising tool to address the dynamic behavior of biological molecules. Recently, Shaikh and Tajkhorshid carried out molecular dynamics (MD) simulations on cASIC1, which provided useful information for the potential binding sites of cations and protons in ASIC1 [12]. However, the current MD methods are limited to address the local movements of proteins. As a complementary approach, normal mode analysis (NMA) [13]–[15] is efficient for predicting the collective dynamics and inherent flexibilities in biological macromolecules. This method has been widely applied in studying the structure (dynamics)-function relationship for several important ion channels, such as the prokaryotic large conductance mechanosensitive channel (MscL) [16], the potassium ion (K^+^) channe1 [17]–[19], and the nicotinic acetylcholine receptor (nAChR) [20]–[23].

In the present study, the dynamic behavior of ASIC1 has been studied on the basis of the crystal structure of cASIC1 using NMA along with complementary mutagenesis and electrophysiological experiments. The NMA revealed complementary twisting motions throughout the receptor, with which the ion channel may undergo an open motion. Further analyses on the motion modes detect a series of collective movements among the subdomains of EC domain, which control and induce the motions of channel pore. Furthermore, the twisting motion modes of the TM domain indicate the probable position of the channel gate. Electrophysiological assays on the human ASIC1a (hASIC1a) mutants of a series of key residues associated with the motions support the computational results. This study provides new information on the intrinsic dynamic behavior of ASIC1 motions associated with the channel opening, enabling us to construct a new model for the gating mechanism of the channel. This model, supported by a number of key experimental observations from others as well as our own, for the first time to our knowledge, provides a clear picture of the correlation between the structural dynamics of ASIC1 and its gating mechanism.

## Results

### The Twisting Motions of TM Domain Disclose the Possible Gate of ASIC1

**Figure 2:**
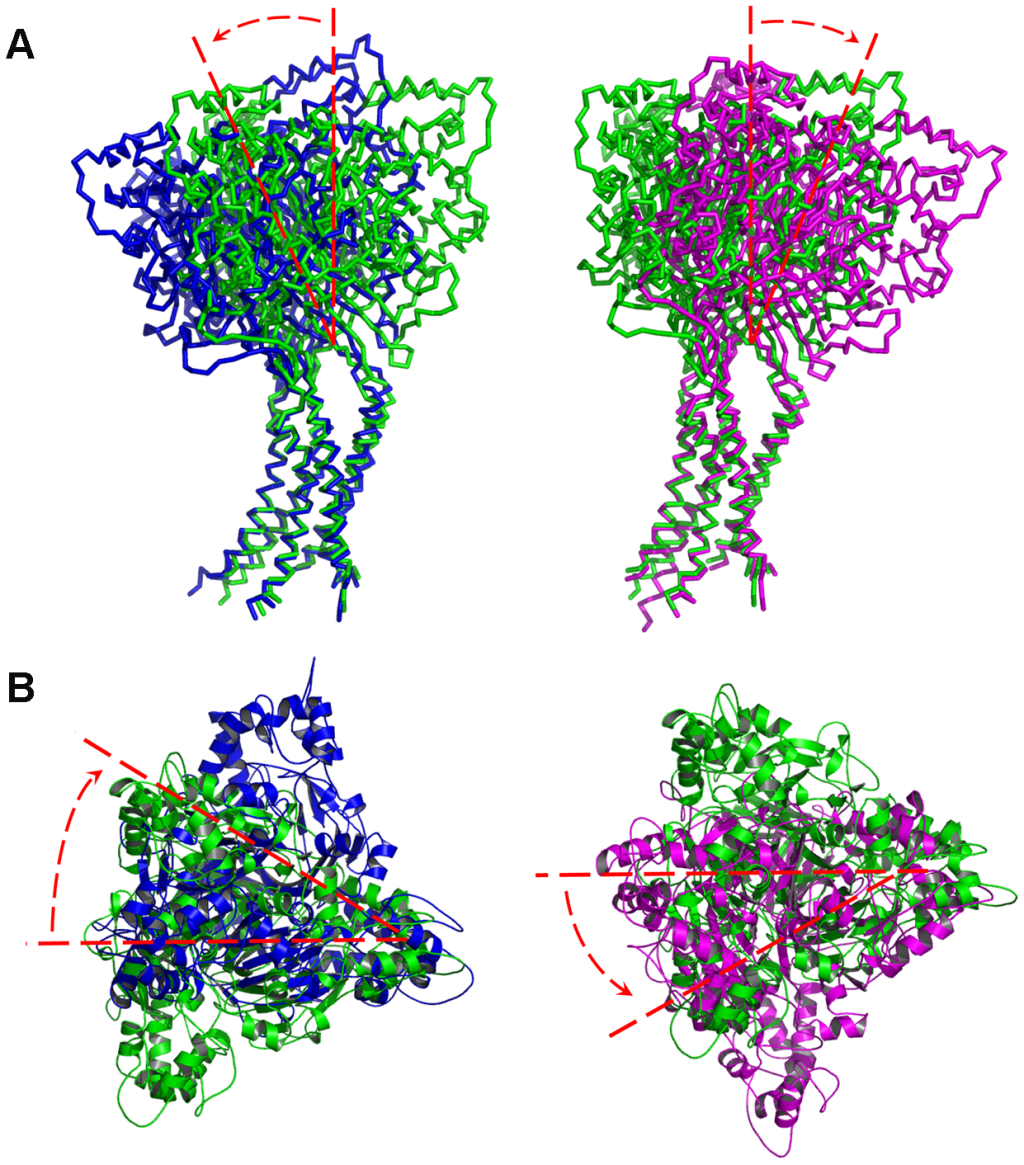
General motions of ASIC1 as detected by NMA. (A) Rocking motion between the EC domains. Two snapshots at harmonic periods one-quarter (blue) and three-quarters (magenta) of mode 2 were superimposed to the initial structure (green) by fitting all the C_α_ atoms of the TM domain. The arrows show the direction of the rocking motions. (B) Rotation of the EC domain around subunit C. Two snapshots at harmonic periods one-quarter (blue) and three-quarters (magenta) of mode 3 were superimposed to the starting structure (green) by fitting all the C_α_ atoms of the TM domain. The arrows indicate the rotation direction of the EC domain.

NMA is a computational approach that can efficiently predict the collective dynamics and inherent flexibilities in biological macromolecules. Accordingly, we used NMA to detect the intrinsic motion modes of ASIC1. First, we examined the low-frequency modes of ASIC1 produced by NMA because they may reflect the global motions of the ASIC1 channel and are often potentially related to biological functions [24],[25]. The 100 lowest frequency modes resulted from NMA were used to describe the overall motions of the entire channel, since these normal modes are sufficient to capture all the collective motions of the ASIC1, as revealed in other proteins [25],[26]. The detailed motions between the essential subdomains (e.g., thumb and finger) are discussed in the following sections and the motions of some important modes are presented in Figures S1 and S2 and Videos S1, S2, S3, S4, S5. In brief, these modes revealed conformational changes that may involve the whole receptor including a rocking motion of the EC domains around the wrist region which connects the EC and TM domains (Figure 2A) and coinstantaneous rotations of the EC and TM domains around subunit C (Figure 2B).

**Figure 3 pbio-1000151-g003:**
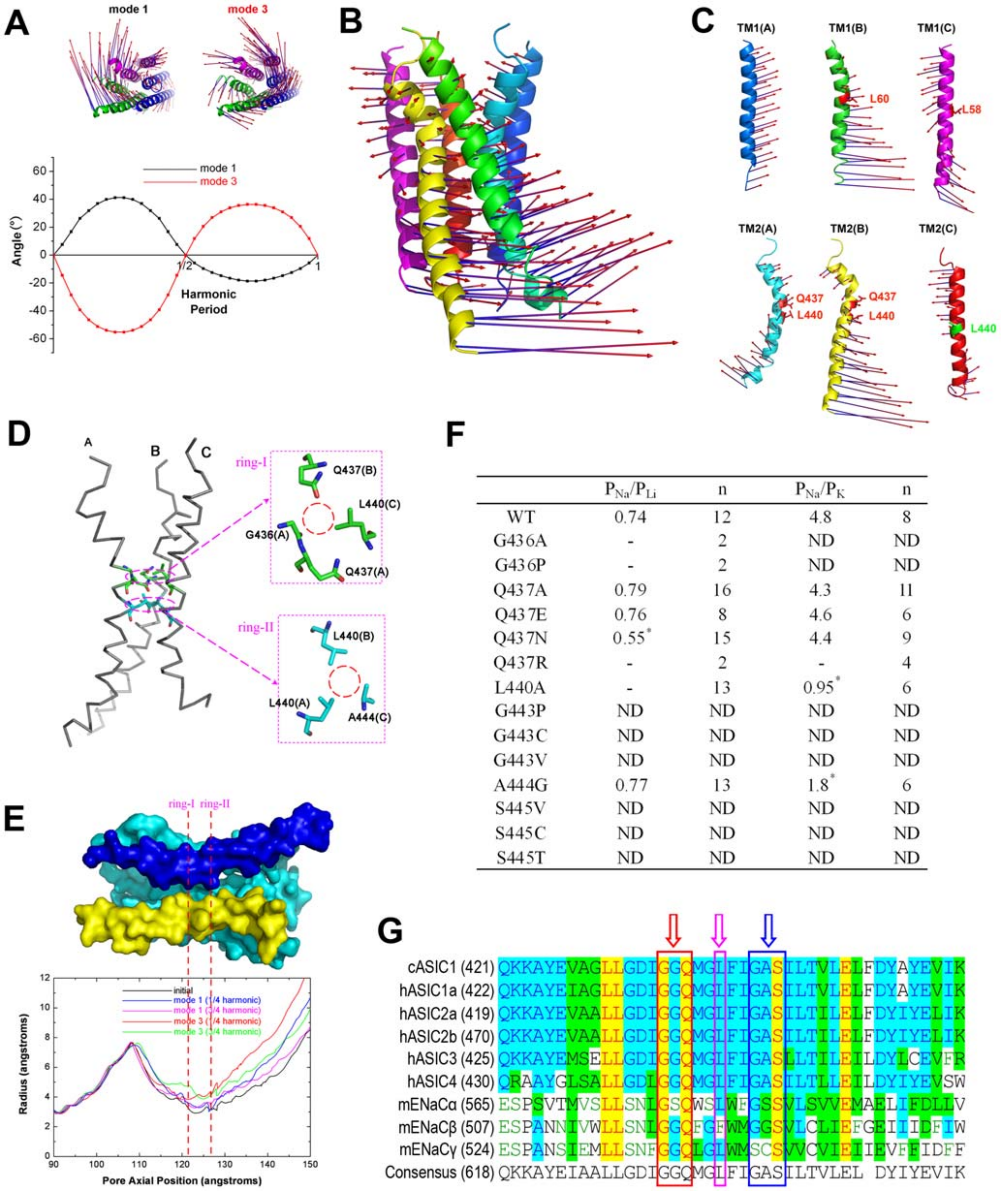
The dynamics of the channel pore and possible position of the gate. (A) General rotation motion of the TM domain in modes 1 and 3 (top). The vectors representing both the amplitudes and directions of the displacements experienced by residues during the conformation changes are mapped onto the TM domain. This is the same for (B) and (C). As indicated by the vectors, the motions can be described approximately as general rotation around the pore axis. (Bottom) The rotational angles of the TM domain along the harmonic period in modes 1 and 3. (B) The side view of the twisting rotation of the TM domain in mode 1. (C) Vector representation of the twisting motion of each individual TM1 or TM2 helix in each subunit (shown in side view). With the exception of TM1(A), the other five helices change the direction of the motion around their own hinges. The hinge is located around L60 for TM1(B), L58 for TM1(C), Q437 and L440 for TM2(A) and TM2(B), and L440 for TM2(C). These motions synthesize a twisting rotation of the whole TM domain as shown in (B). (D) A schematic representation of the channel pore structure. The residues that compose the bottleneck can be divided into two layers (rings). The first layer (ring-I) is composed of G436 (A), Q437 (A), Q437 (B), and L440 (C). The second layer (ring-II) is composed of L440 (A), L440 (B), and A444 (C). The residues composing ring-I and ring-II are the same as those act as the hinges of the twisting motion of the three TM2 helices (C). (E) Profile of the pore radius of modes 1 and 3 along the pore axis. The TM helices are shown in surface representation to highlight the pore shape. For clarity, the TM1 helix of subunit C was removed. The TM2 helices of subunits B and C are shown in blue and yellow, respectively. TM1 and TM2 helices of subunit A and TM1 helix of subunit B are colored in cyan. The colored lines represent the pore radius profiles of four structural snapshots as compared to that of the crystal structure of cASIC1 (PDB entry 2QTS). The bottleneck of the pore is the region between the two dashed lines (ring-I and ring-II in [D]). The radius profiles for modes 1 and 3 show dilation of the TM segment. (F) Relative permeability ratio of Li^+^, Na^+^, and K^+^ in CHO cells transected with WT or mutated ASIC1 channel. The values were calculated as described in “Data Analysis.” -, no response to pH 5.0; ND, not determined. (G) Amino acid sequence alignments for the pore regions of ASICs from chicken (cASIC1, GenBank [http://www.ncbi.nlm.nih.gov/Genbank] ID: 63054900) and human (hASIC1a, 21536349; hASIC2a, 1280439; hASIC2b, 21739677; hASIC3, 3747101; and hASIC4, 8346834), and ENaCs from mouse (mENaCα, 149361431; mENaCβ, 6755411; mENaCγ, 118130196). Open arrows and boxes indicate the conserved residues around the bottleneck. These conserved residues are divided into three tracts: GGQ tract (red box), L tract (magenta box), and GAS tract (blue box).

NMA revealed that the most relevant modes of ASIC1 to its gating mechanism might be modes 1 and 3, which undergo similar motions. In addition to the rock and rotation of the EC domain (Figure 2), the six TM helices underwent a concerted global rotation in both clockwise and anticlockwise directions, as indicated by the rotating angles along the harmonic period (Figure 3A). However, the whole TM domain did not rotate in a simple manner; instead, it may adopt a twisting rotation during the conformational change. As illustrated by the motion of the TM domain in mode 1 (Figure 3B), the direction of the motion as well as the displacement of the movement for different regions along the pore axis (e.g., top and bottom) were different. As shown in Figure 3C, with the exception of TM1(A), the other five helices may undergo twisting motions, changing the direction of their motion around the hinges: L60 for TM1(B), L58 for TM1(C), Q437 and L440 for TM2(A) and TM2(B), and L440 for TM2(C). As a result, the twisting motions of the five helices synthesize a twisting rotation for the whole TM domain as represented in Figure 3B. This result implies that the region near the hinge for the twisting rotation of TM domain is important for controlling the gating of the ASIC1. This hinge is located between G436 and L440 of the TM2 helices (Figure 3D). Because of the asymmetric nature of the ASIC1 structure, the innermost residues of three TM2 helices around the hinge form two rings (designated ring-I and ring-II hereinafter). Ring-I is composed of G436(A), Q437(A), Q437(B), and L440(C), and ring-II consists of L440(A), L440(B), and A444(C) (Figure 3D). Similar to the nAChR [23], the position of the gate of ASIC1 is possibly located near the hinge of the pore. Accordingly, we monitored the radius profiles along the pore axis for each motion mode. Indeed, the channel pore has a bottleneck around the hinge (∼125 Å) (Figure 3E). Rather, the radius profiles for modes 1 and 3 display a visible opening of the channel pore (Figure 3E), suggesting that the twisting motion of TM tends to increase the width of the entire pore. This result indicates that the structure of the closed, desensitized state of ASIC1 intrinsically tends to undergo a twisting motion to open the gate. The hinge sharply divides the pore into two portions: a top segment (110–125 Å) and a bottom segment (125–150 Å). The displacements of the channel diameters for the bottom segment are larger than those of the top, suggesting that the top segment may play a larger role in ion selectivity. Moreover, the motion of mode 3 indicates that the diameter of the bottleneck may maximally increase ∼2 Å (Figure 3E). This displacement in pore diameter is significant for the function of the channel, because it has been suggested that even relatively minor displacements in the gate area can trigger the functional transition of the pore from closed to open [27].

After predicting the positions of the ASIC1 channel gate using NMA, we investigated the molecular basis of the ASIC1 gating further using mutagenesis and electrophysiological experiments. Using human ASIC1a (hASIC1a), we constructed eight mutants around the putative gate position of ring-I and ring-II (Figure 3D): G436A, G436P, Q437A, Q437E, Q437N, Q437R, L440A, and A444G. The profiles of the inward currents elicited by acidic solution in the wild-type (WT) ASIC1 and its mutants are shown in Figure S3. Three mutants (G436A, G436P, and Q437R) resulted in nonfunctional channels as no current was detected in response to pH 5.0 in these mutants, suggesting that G436 and Q437 are key determinants for ASIC1 function. In addition, several mutations (Q437N, L440A, and A444G) altered the reversal potentials of acid-induced currents (Figure S4; Table S1). Consistent with the altered reversal potentials, the relative Na^+^, Li^+^, and K^+^ permeability of these mutated channels were also significantly shifted (Figures 3F and S4). Interestingly, whereas the reversal potentials of the Q437A, Q437E, and Q437N mutants were relatively unchanged (i.e., reflecting unaltered Na^+^/K^+^ selectivity) with respect to the WT channel under standard intra- and extracellular ion compositions (Table S1), the selectivity of Na^+^ over Li^+^ (*p*_Na_/*p*_Li_-value) of Q437N mutant decreased significantly (Figure 3F). Furthermore, replacement of Q437 with a positively charged residue Arg (Q437R) caused the loss of function of the mutated channel presumably because of the repulsion between monocations and the Arg side chain in the pore region (Figure S3). These results suggest that Q437 is critical for ion passage. Besides G436 and Q437, we found that two additional residues L440 and A444 affected the ASIC1 gating markedly because the reversal potential and the selectivity of Na^+^ over K^+^ (*p*_Na_/*p*_K_-value) for both L440A and A444G mutants decreased dramatically (Figure 3F). We attribute this phenomenon to the enlargement of ring-I or ring-II due to L440A and A444G replacement, and the mutated channels pass more K^+^ than Na^+^ with respect to WT channel. These results indicate that L440 and A444 play important roles in ion selectivity and gating of ASIC1.

It should be emphasized that the identified key residues contributing to the possible gate of ASIC1 are mostly conserved among the superfamily of ASICs as indicated by the sequence alignment (Figure 3G). On the basis of electrophysiological studies, previous studies suggested that G587 and S589 in the α-subunit are key residues to define the selectivity filter of epithelial sodium channel (ENaC) [28]–[31]. In ASIC1 these two residues correspond to G443 and S445, respectively. Mutations of G443C, G443V, G443P, S445C, S445T, and S445V all resulted in a nonfunctional ASIC1 (Figure S3). Interestingly, as mentioned above, mutating A444, a residue located between G443 and S445, to Gly, resulted in an intact ASIC1 with significantly altered ion selectivity (Figure 3F). However, the corresponding residue of A444 in ENaC (S588 of α-subunit) does not affect the ion selectivity of ENaC [29]. Thus the region for ion selectivity in ASIC1 may be the same as that in ENaC, but the residue contribution is different between these two ion channels (Figure 3G). In addition, we found that L440 also played a critical role in the ion selectivity of ASIC1 (Figure 3F). Nevertheless, the corresponding residue of L440 in ENaC (L584 of α-subunit) does not contribute to the ENaC ion selectivity [31]. We attribute this difference to the distinct “gating” mechanisms of ASIC1 and ENaC. In fact, one striking difference between these two channels is that ENaC is constitutively active (i.e., without a functional “gate”) [3], whereas ASIC1 opens following channel gating by agonist (proton) binding. Taking together, we conclude that the region around ring-I and ring-II (Figure 3D) may undergo a substantial conformational change that is coupled to channel gating and constitutes an important regulatory region of ASIC1 function.

### Collective Motions of the β Turn and TM Induce the Opening of the Channel Pore

**Figure 4 pbio-1000151-g004:**
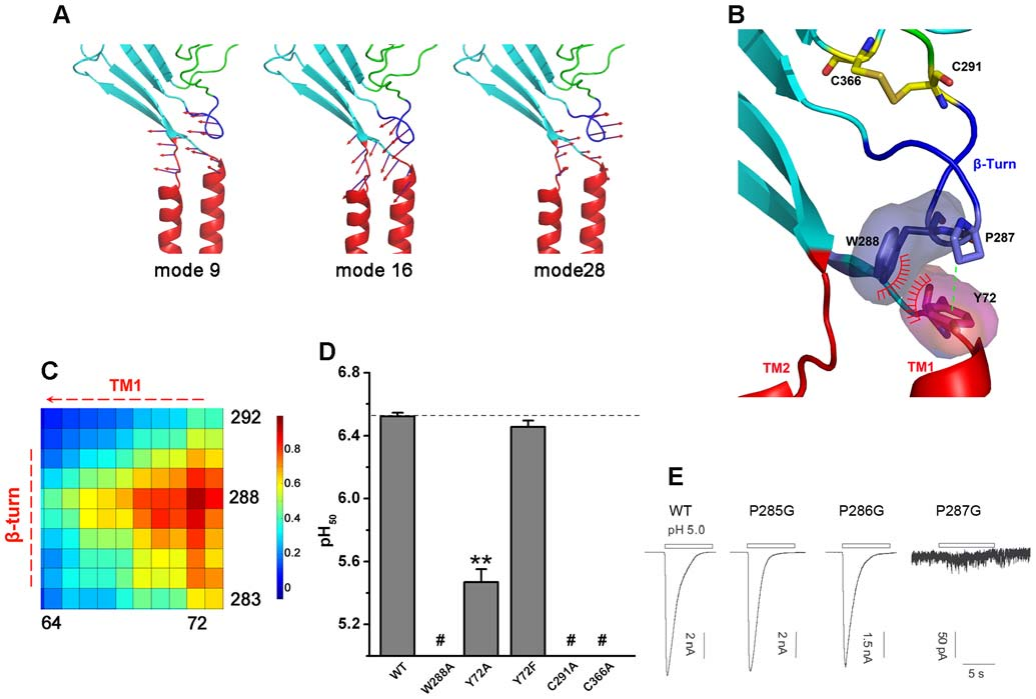
Collective motions between the β turn and TM domain are essential for channel gating. (A) Collective motions between the β turn and the TM domain. These two subdomains show collective motions in most of the NMA modes. The motions in modes 9, 16, and 28 are shown as examples to demonstrate the collective motions. The vector arrows represent the amplitude and direction of the displacement experienced by each residue around the β turn, clearly illustrating the collective motions between the β turn and the TM domain. (B) Essential interactions between the β turn and the TM domain. Y72 interacts with W288 via π–π stacking and with P287 via C-H–π hydrogen bonding; these residues are displayed in stick mode. The C291–C366 disulfide bond that is important in stabilizing the β-turn conformation is also represented in stick mode. (C) Correlation map for the motions experienced by residues around the β turn. Correlated motions are derived from the full set of normal modes. The movements of Y72 show a high correlation with the movements of W288 and P287, with correlation coefficients (*C*_ij_ value) of 0.92 and 0.82, respectively. (D) pH_50_ values of mutations around the β turn of hASIC1a. Data are presented as mean±standard error of the mean (SEM) (shown as error bars). Student's *t*-test analysis shows that the differences in reversal potentials for the WT and mutant channel are statistically significant (all *p*<0.0001 versus WT [**]). Mutants that abolished acid-induced current are indicated with #. (E) Representative traces of currents for the P285G, P286G and P287G mutants. Similar results were obtained from five other recordings. The bars above the recordings indicate the durations of pH 5.0 application.

After detecting the motions of the pore and the probable gate position of the channel, we asked how the channel pore undergoes the twisting-to-open motion. For this purpose, we examined the relationship between conformational changes of other parts of the EC and the TM domain. Structurally, the β1 and β12 strands are connected to TM1 and TM2, respectively; the β9 and β10 strands are linked to the thumb subdomain; β1, β12, β9, and β10 form a metacarpal plane (M-plane) (Figure 1). There is a loop between β9 and α5, and the β turn of the loop interacts with the TM domain via directly interactions of Y72 with W288 and P287 (Figure 4B). Accordingly, the motions of M-plane and the loop should be essential to the conformational changes of the TM domain. To test this idea, we closely examined the motions of the M-plane and the loop between β9 and α5. Indeed, the motions of the M-plane in most of the modes are associated with the motions of the channel pore. For example, the shearing vibration of the M-plane in mode 3 induces TM1 and TM2 helices to undergo twisting movements, and the bending of the M-plane in mode 11 triggers a swinging motion to the TM domain (see Videos S1 and S2). Intriguingly, the motions of the TM domain are coupled with the motions of the β turn (Figure 4A). The synchronous motions of the β turn along with the TM helices are possibly due to the strong hydrophobic interactions of Y72 with W288 and P287 (Figure 4B), indicating the importance of these residues in the gating of the channel. In addition, on the basis of the NMA modes, we also derived the cross-correlation map, which displays the correlations between the movements of different residues (Figure S1). The cross-correlation map also shows that the movement of Y72 correlates strongly with that of W288 and P287 with correlation coefficients of 0.92 and 0.82, respectively (Figure 4C).

Key interactions between the β turn and the TM domain important for the gating mechanism were further characterized by electrophysiological experiments. To this end, we designed mutations to respectively disrupt the π–π stacking interaction between Y72 and W288, the C-H···π hydrogen bonding [32] between P287 and Y72, and the disulfide bond between C291 and C366 (Figure 4B). We found that the W288A mutation abolished the opening of the channel (Figure 4D), and the Y72A mutation greatly decreased the pH sensitivity of the channel. These results suggest the importance of the hydrophobic (mainly π–π stacking) interaction between W288 and Y72 for controlling the channel gating (Figure 4B). To further test this hypothesis, we investigated the pH sensitivity of the Y72F mutant, which may keep the π–π stacking intact. As expected, the electrophysiological characteristic of this mutant was unchanged (Figure 4D).

The C-H···π hydrogen bond between P287 and Y72 represents another important interaction that may be responsible for the collective motion of the β turn with the TM domain. In addition, because P287 is located at the lip of the β turn, it may play a structural role in stabilizing the β-turn conformation and likely affect channel gating. As predicted, the P287G mutation abolished the channel opening capacity (Figure 4E). On the other hand, P285G and P286G mutants were unaffected, suggesting that these two residues are not important for the channel gating. Finally, we studied the disulfide bond (S–S bond) interactions between C291 and C366 (Figure 4B). In addition to stabilizing the conformation of the β turn, this disulfide bond is a linkage between the β turn and the β10 strand, which is a bridge that conducts the motions of the finger and knuckle to the β turn as will be discussed in next section. Hence, we hypothesized that this disulfide bond may also play a role in the channel gating. To test this idea, electrophysiological characterizations were performed on C291A and C366A mutants. Both C291A and C366A mutations resulted in termination of the channel opening activity (Figure 4D).

Of note, the importance of the interaction between Y72 and W288 to the channel gating was also addressed by Li et al. [33]. The result was reported online during the reviewing process of this manuscript.

### Collective Motions of the Thumb and Finger Correlate with Channel Gating

**Figure 5 pbio-1000151-g005:**
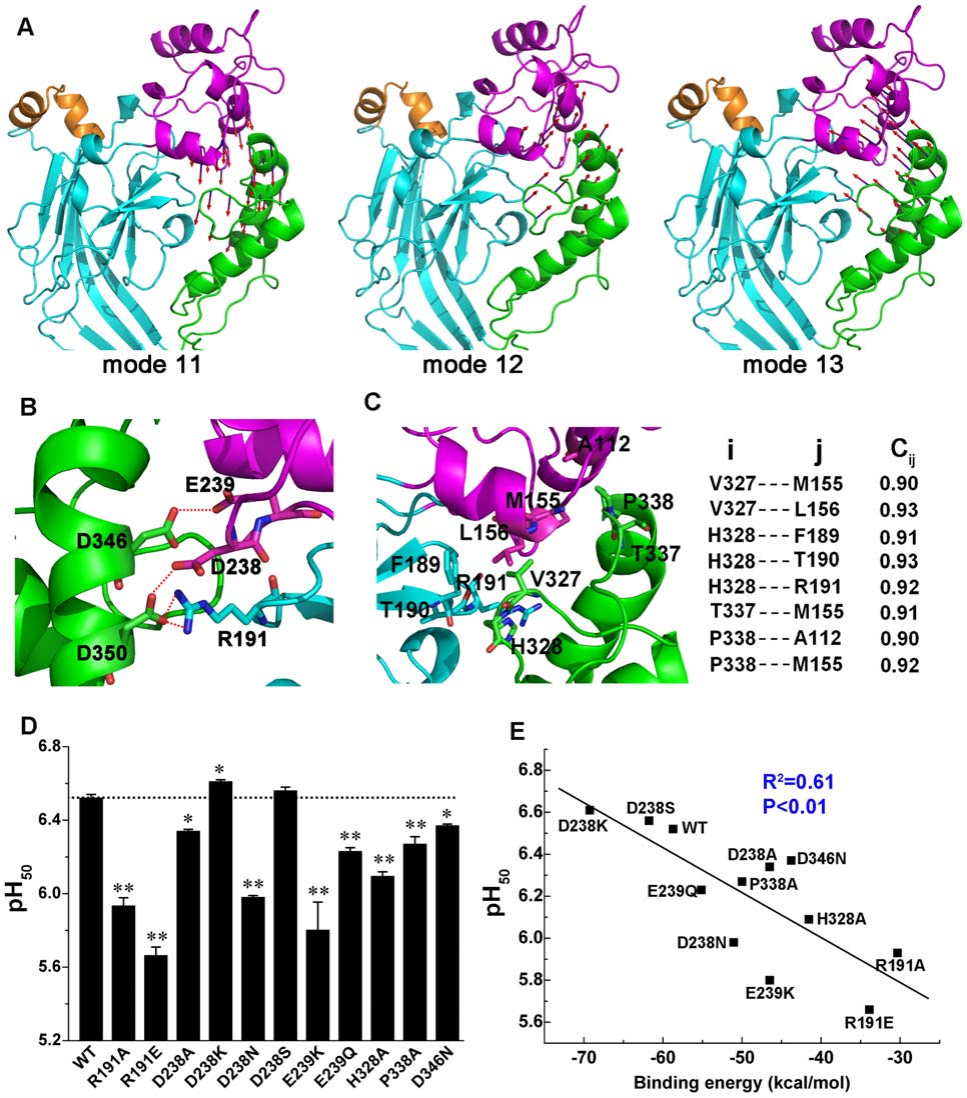
Correlation between the collective motions of thumb with finger and the channel gating. (A) Cooperative motions of the thumb (green) with finger (magenta). These motions exist in most of the NMA modes. The motions in modes 11, 12, and 13 are shown as examples of such collective motions. The vector arrow represents the amplitude and direction of the displacement experienced by each residue on the interface between the two subdomains. The arrows mapped onto the structure clearly display the collective motions between the thumb and finger. (B) Hydrogen bonds between the thumb and finger domains. Key residues include and are represented in stick view. The dashed red lines represent hydrogen bonds. (C) Close-up view of the hydrophobic interface between the thumb and finger (left). The cross-correlation coefficients (*C*_ij_) of some important residue pairs between thumb (i) and finger (j) (right). (D) pH_50_ values for the WT channel and mutants (R191A, R191E, D238A, D238K, D238N, D238S, E239K, E239Q, H328A, P338A, D346N) that were designed to test key hydrogen bonding and hydrophobic interactions. Data are from five and three separate patches for kinetic and dose–response experiments, respectively. Student's *t*-test analysis shows that the differences in pH_50_ for the WT and mutant channels are statistically significant. *, *p*<0.05 versus WT; **, *p*<0.001 versus WT. (E) A linear correlation exists between binding free energies (ΔG_binding_) of thumb and finger domain interactions and their corresponding pH_50_ values for the WT and the mutant channels described in (D). The binding free energies between the thumb and finger of the WT and all the mutants were calculated using the MM-PBSA method encoded in AMBER, version 9.0 [39]. The detailed computational procedure is described in the Text S1.

The crystal structure of cASIC1 suggests that one end of the β turn links to the thumb domain and the other end connects to the knuckle domain through the β9 strand; the S–S bond between C291 and C366 also plays an important role to the interaction between the β turn and the M-plane. In addition, the TM1 and TM2 helices connect with the finger and knuckle domains through the β1 and β12 strands, respectively (Figure 1). This structural arrangement indicates that the EC domain may communicate with the TM domain and β turn through a series of collective motions, implying that the collective motions of different regions in the EC domain are possibly relevant to the channel gating. Accordingly, we analyzed the motion modes of the EC domain. Interestingly, all motion modes revealed that the thumb always moves correlatively with the finger (Figure 5A). This result suggests that channel gating may be facilitated by the attractive forces between the thumb and finger domains. Essential hydrogen bonding and hydrophobic interactions between the thumb and finger are shown in Figure 5B and 5C, respectively. To test this hypothesis, we first disrupted several pairs of hydrogen bonds (H-bonds) and electrostatic interactions between the thumb and finger via site-directed mutagenesis, including D238···D350, E239···D346, and R191···D350 pairs, which are identified by Jasti et al. as tentative proton binding sites (Figure 5B) [11]. Abolishment of the R191···D350 and E239···D346 H-bonds by substituting R191 with Ala, E239 with Gln or Lys, and D346 with Asn, and the D238···D350 H-bond by substituting D238 with Ala or Asn had profound effects on the pH_50_ (pH of half-maximal activation) of the acid-induced currents (Figure 5D); all of these mutations reduced the pH_50_ values of the ASIC1 (Figure 5D). Similar effects had been observed with the D346N mutation by Jasti et al. [11]. Binding free energy (Δ*G*_binding_) calculations indicate that all these mutations decrease the binding affinity between the thumb and finger (see Discussion, Figure 5E, and Table S2). To firmly establish the importance of the attractive interaction between the thumb and finger to the opening of the gate, we designed another two mutants, D238K and D238S, which might enhance the interaction between the thumb and finger. Binding free energy calculations are consistent with this notion, as the Δ*G*_binding_ values between the two subdomains for these two mutations are respectively reduced by ∼10.0 and 3 kcal/mol relative to WT (Figure 5E; Table S2). Consistently, these mutations also led to higher pH_50_ values than that of the WT channel (Figure 5D).

Hydrophobic interactions are another dominant component to the collective motion between the thumb and finger. NMA results indicate that the pairs of hydrophobic interaction between these two subdomains move together with a high correlation as their cross-correlation coefficients (*C*_ij_) are larger than 0.9 (Figure 5C). This suggests that mutations that decrease the hydrophobic interaction between these two subdomains would cause the channel to respond to a lower pH. We thus mutated H328 and P338 to Ala to weaken the hydrophobic interaction between the thumb and finger domains. The electrophysiological results are consistent with the computational predictions, i.e., both mutations decreased the pH_50_ values (Figure 5D). Remarkably, the calculated binding free energies between the thumb and finger for the WT channel and all its mutants correlate well with the pH_50_ values with a high correlation coefficient, *R* ^2^ = 0.61 (Figure 5E). These results, together with our NMA analysis, strongly support that the collective motions between the thumb and finger are of significance to the channel gating.

### Deformation Pathway and Flexibility of ASIC1 Related to Channel Gating

**Figure 6:**
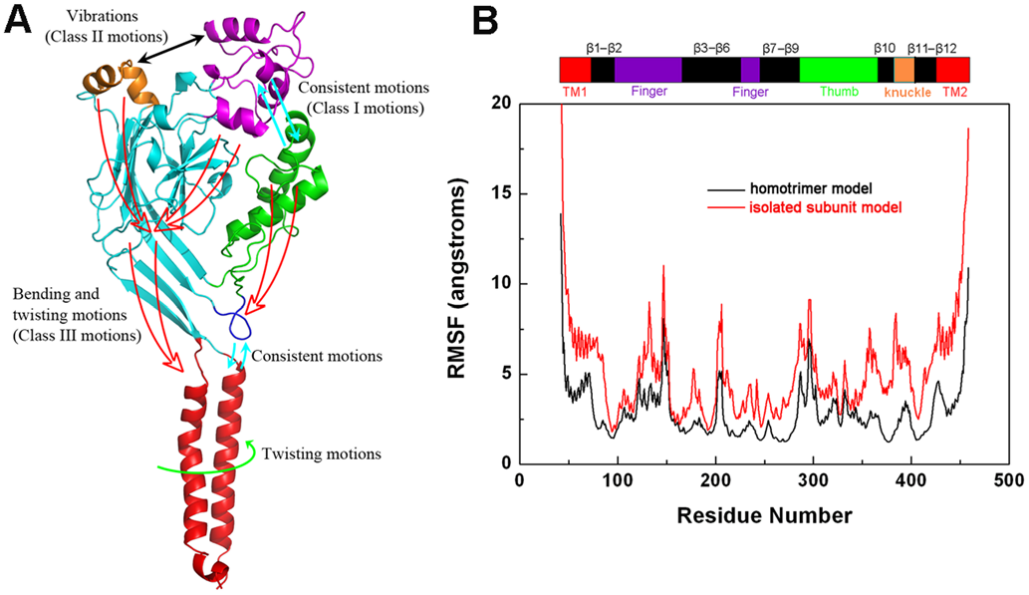
Deformation pathway and flexibility of ASIC1. (A) Possible deformation pathways related to gating of ASIC1. Monomer is shown in cartoon representation with subdomains colored in green (thumb), magenta (finger), cyan (palm), orange (knuckle), blue (β turn), and red (TM). Three classes of movements within EC domain have been identified by NMA calculations: class I (consistent motions of thumb with finger, cyan arrows), class II (vibrations between finger and knuckle, black arrows), and class III (bending and twisting motions of the M-plane, red arrows). These motions are conducted to the TM domain through the M-plane and the consistent motions between β turn and TM. The green arrow indicates the twisting motions within TM domain. (B) The RMSF profiles for ASIC1 as a homotrimer (black line) versus its individual state (red line). ASIC1 subdomains are denoted at the top and correspond to the residue numbers listed along the horizontal axis.

Our studies revealed collective motions that occur amongst the subdomains of the EC domain, so we sought to map the deformation pathway related to channel gating because of its importance in understanding the overall function of ASIC1. In addition to the collective motions between the β turn and TM domain and between the thumb and finger domains mentioned above, bending and swing vibrations between the finger and knuckle were also revealed by NMA (Figure S2). These motions lead to bending and twisting motions of the M-plane (β1, β12, β9, and β10), which further evoke different motions of the TM domain (Videos S3, S4, S5). Accordingly, the NMA modes clearly show the deformation pathway for domain motions: collective motions of thumb with finger (class I motions) couple with the vibrations between finger and knuckle (class II motions), which further associate with the bending and twisting motions of the M-plane (class III motions) (Figure 6A). Both class I and class III motions are able to trigger the TM domain to undergo rotation and twisting motions and the β turn to engage in swinging motions; the latter movement enhances the motions of the TM domain through noncovalent interactions (Figure 4B). This deformation pathway demonstrates the inherent structural flexibility of ASIC1 for implementing their functions and also implies the functional importance of the EC domain of the receptor.

To further understand the dynamic behavior of each subunit in isolation versus its homotrimer conformation and their relation to gating, NMA was also performed on the monomer using the structure of the subunit taken from the X-ray crystal structure of cASIC1 [11]. Again, the first 100 lowest frequency normal modes were obtained. Most of the motions of the monomer are, in general, similar to those of each subunit within the trimer, suggesting that the intrinsic properties of each subunit determine the receptor's motions, which further control the gating of the whole channel. Figure 6B shows the differences in flexibility between the monomer treated as an isolated subunit (red trace) versus part of the trimer (black trace), as shown in the profile of the root-mean-square-fluctuations (RMSFs) from the 100 lowest frequency normal modes. Here, we only use subunit A to discuss the differences, because similar results were obtained for subunits B and C. As shown in Figure 6B, the flexibility of each domain is restricted in the trimer compared to the monomer. In both the monomer and as part of the trimer, the tips of the thumb and finger are two of the most flexible portions. This result is consistent with the harmonious motions of these two domains, demonstrating again the importance of their motions in gating. In fact, Jasti et al. have hypothesized that the cleft between the thumb and finger contains an acidic pocket for sensing acidic conditions (i.e., proton levels) [11]. Moreover, our electrophysiological experiments on the mutants of the residues along the acidic pocket also suggest that the motions of these two domains are linked to the physiological function of this protein (Figure 5). Another region that displays pronounced flexibility in the individual monomer is the knuckle domain. Although the mobility of the knuckle is restricted in the trimer, its tip still shows high fluctuation (Figure 6B), enabling vibration motions between the knuckle and finger. Other regions showing high fluctuation are the TM1 and TM2 helices, but their flexibility is also restrained in the trimer, especially the TM2 helix (Figure 6B). Remarkably, the RMSF profiles for both TM1 and TM2 helices in the trimerized channel form inverted parabola-like curves, indicating that the two ends are more mobile than the middle, and the flexibility of the hinge position is seriously restricted (Figure 6B). This result is consistent with the twisting motion modes and site-directed mutagenesis results (Figures 3 and S3; Table S1). When existing as part of the trimer, most of the β strand's flexibility is also reduced, but they still undergo local motions induced by the motions of thumb, finger and knuckle. The collective evidence obtained by the NMA results and the flexibility map suggest that the deformation pathway involves the following domain motions: thumb, finger, and knuckle are activists of the receptor, their dynamic behaviors concomitantly propagate to the palm, leading β1, β9, β10, and β12 to undergo bending and twisting motions and the β turn to undergo a swinging vibration. These motions are further transmitted to the TM domain, triggering a twisting motion that opens the channel pore.

## Discussion

While X-ray crystal structures of the closed, desensitized-like state of the cASIC1 channel has been determined recently [11], some important issues of the gating mechanism of the ASIC channels are still unknown. Here, we use a combination of computational simulations together with electrophysiological measurements to investigate the relationship between the inherent dynamics of ASIC1 and its gating mechanism. The deformation pathway derived from the NMA calculations is compatible with our site-directed mutagenesis and electrophysiological characterizations, providing a plausible model for the conformational changes underlying the gating mechanism of ASIC1.

We have carried out normal mode analyses on both trimer and monomer forms of cASIC1 by using the X-ray structure, representing the closed-form, as the starting model. The first 100 lowest frequency modes were obtained and analyzed for each of the starting models. The NMA modes are of particular interest because they reveal intrinsically dynamic motions of ASIC1 that are essential to the gating process and function of the receptor.

The lowest frequency modes of each subunit in isolation (monomer) and in the trimer revealed the most flexible portions of cASIC1. Each subunit is more flexible in the isolation state than in the trimer, suggesting that the trimerization of the receptor restrains the mobility and plasticity of the subunits (Figure 6B). Still, the thumb, finger, and knuckle of the EC domain maintain enough flexibility to carry out the receptor's function in gating (Figure 6B). The TM domain shows distinct flexibility. In general, TM1 is more flexible than TM2, and the two ends of both TM helices are more flexible than their middle parts, resulting in parabola-like curves of RMSF profiles for these two helices, of which the valleys are located around the hinge point of the channel pore (Figure 6B). This indicates that the flexibility of the receptor is amenable to the requirement of gating.

We investigated the holistic motions of the EC domain by analyzing the motion modes. The EC domain as an entire body may undergo rotation around the pore axis and swing round the wrist region of the receptor. For example, the EC domain in mode 2 displays a rocking vibration (Figure 2A), and in modes 1 and 3 experiences a gyroscopic precession like a rotating peg-top, as viewed by superposing the TM domain (Figure 2B). These motions trigger the TM domain to undergo a twisting motion, which is possibly related to the channel gating as demonstrated by the mutagenesis and electrophysiological characterization. In addition, the rigid body motions of EC not only can trigger the conformational changes of the TM domain, but also drives the subdomains within EM to undergo further motions specific to gating. In particular, the thumb and finger domains may undergo swinging and rocking motions (Figure 5A); in addition, bending and swinging vibrations were also found between the finger and knuckle domain (Figure S2). Collectively, these motions provoke the M-plane and β turn to undergo vibration and swinging motions, which directly stimulate the functional motions of the TM domain (Figure 6A). Accordingly, the inherent flexibility and dynamics of ASIC1 are tightly associated with the channel gating.

Two important findings that were significant to the gating mechanism were uncovered amongst the normal modes of EC domain. The first is the consistent motions between thumb and finger domains (Figure 5A). This result indicated the vital role of the attractive interaction between these two subdomains in the gating process. Indeed, a series of mutations and corresponding electrophysiological measurements have confirmed this hypothesis (Figure 5D and 5E). Another point is that the β turn always moves concomitantly with the motions of the TM domain, demonstrating that the interaction between the β turn and TM domain dominates the gating (Figure 4A–4C). This conclusion has also been verified by our mutagenesis and electrophysiological experiments (Figure 4D and 4E).

NMA also revealed a notable twisting to open pore motion located in the TM domain, which is directly associated with channel gating (Figure 3). Most importantly, the twisting motions of the TM helices are evoked by the motions of the EC domain (Figure 6A). Meanwhile, the hinge of twisting motions positions around the bottleneck of the channel pore (ring-I and ring-II, Figure 3D) suggests that this region (Q437–A444) might be the gate of the channel. This computational prediction has also been validated by using a series of mutagenesis experiments (Figures 3F and S3; Table S1). Clearly, the architecture of ASIC1 is organized to allow for communication between the EC and TM domains through a deformation pathway, which triggers the gating process of the channel (Figure 6A).

### Possible Driving Forces for Stimulating Motions of the Thumb and Finger

The function of the ASIC large EC domain in the gating mechanism remains to be elucidated. Results of this study indicate that motions of all subdomains and regions of the EC domain may collectively stimulate the motions and conformational changes of the TM domain, affecting the shape of the channel pore (Figure 6A). The dynamic pathway seems to be associated with the function of EC domain, which raises an intriguing question: what is the driving force that triggers these motions? A large body of evidence has indicated that the channel opens in response to hydrogen ions, allowing sodium ions to pass into the cell [1],[2]. The recently determined X-ray crystal structure of cASIC1 [11] and MD simulation [12] suggest that H^+^ ion may bind to an acidic pocket between thumb and finger subdomains. This process provides the energy to trigger movement of the EC domains; however, how the H^+^ binding would drive such events is still unclear. Here, we provide a possible explanation based on our NMA and mutagenesis experiments.

On the basis of the holistic motion modes of the EC domain and the consistent motion of thumb and fingers to the channel gating described above, we hypothesize that the initial driving force for the EC domain movements is the attraction between thumb and finger (Figure 6A). H^+^ binding to the acidic pocket enhances the interaction between these two domains, which heightens their intrinsic motion during gating process. This hypothesis has been verified by a series of mutations and electrophysiological determinations: mutations that either abolish the H-bonds or weaken the hydrophobic interaction between these two subdomains shift the dose–response curve to a lower pH region and decrease the pH_50_ values; and the mutations of D238K and D238S that might enhance hydrogen bonding between the thumb and finger shift the dose–response curve to the higher pH region and increase the pH_50_ (Figure 5D and 5E). Theoretical calculations are consistent with these results: removing the H-bonding interactions and decreasing the hydrophobic interactions between thumb and finger domains decrease the binding free energy between these two domains whereas the D238K and D238S mutants, which strengthen the hydrogen bonding interaction, increase the binding affinity between the two subdomains. Moreover, the binding free energies correlate well with the pH_50_ values (Figure 5E). This computational result demonstrates again that the attractive interaction between thumb and finger might be a driving force to channel gating.

### Dynamic Mechanism for the Proton-Activated Gating of ASIC1

The current study shows that ASIC1 exhibits an intimate connection between the intrinsic structural dynamics and the gating process. On the basis of the NMA results and related mutagenesis and electrophysiological experiments, we propose a dynamic mechanism for the proton-activated gating of ASIC1. The first step of the mechanism is the binding of H^+^ to the acidic pocket [11]. In contrast to an earlier hypothesis that was raised, on the basis of inspection of the crystal structure of cASIC1 [34], we believe that H^+^ binding does not displace the thumb during gating, but instead enhances the binding affinity between thumb and finger through strengthening the H-bonds formed between acidic residues. This conclusion is based on the collective evidence gathered from the mutagenesis and electrophysiological measurements as well as binding free energy calculations on the WT channel and mutants (Figure 5). Thus, H^+^ binding induces thumb and finger domains to move close to each other, thereby initiating and magnifying a series motions along the intrinsic deformation pathway of the receptor (Figure 6A). These motions trigger conformational changes of the TM domain, which provoke the TM domain to undergo a twisting motion to open the gate. This mechanism is clearly of general evolutionary significance of ASIC. The hand-like structure of the monomer and the chalice-like architecture of the entire receptor provide an elegant solution for controlling the gating mechanism of ASIC.

## Materials and Methods

### NMA

The atomic coordinates for the crystal structure of cASIC1 (Protein Data Bank [http://www.rcsb.org/pdb] entry 2QTS) [11] was used as the starting structure in a series of computational simulations and calculations. NMA was conducted using the web server developed by Delarue et al. (http://lorentz.immstr.pasteur.fr/nomad-ref.php) [35],[36]. During the NMA simulations, the single-parameter Hookean potential, a simplified all-atom potential [35], was used (Equation 1),
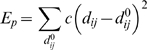
(1)where *d*_ij_ is the distance between two atoms *i* and *j*, 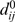 is the distance between the atoms in the 3-D structure, *c* is the spring constant of the Hookean potential (assumed to be the same for all interacting pairs), and *R*_c_ is an arbitrary cut-off. In this study, *R*_c_ was set to be 10 Å.

RMSF of each atom from the nontrivial modes and frequencies was calculated using the method of [37],
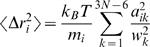
(2)where *m*_i_ is the mass for atom *i*; *ω*_k_ is the vibration frequency of mode *k*; *a*_ik_ is the *i*th components of the *k*th eigenvector.

Cross-correlations (*C*_ij_) of atomic motion were computed with the modes and frequencies derived from the NMA using Equations 3 and 4 [37],
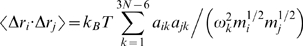
(3)

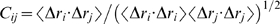
(4)where *m*_i_ and *m*_j_ are the masses for atoms *i* and *j*; *ω*_k_ is the vibration frequency of mode *k*; *a*_ik_ and *a*_jk_ are the *i*th and *j*th components of the *k*th eigenvector.

### Cell Culture and Transfection

All constructs were expressed in CHO cells. Transient transfection of CHO cells was carried out using Lipofectamine 2000 (Invitrogen). Electrophysiological measurements were performed 24–48 h after transfection. The cDNA encoding hASIC1a was a generous gift from Jun Xia (The Hong Kong University of Science and Technology, Hong Kong, China).

### Solution Preparations

The incubation solution contained the following components (in mM): 124 NaCl, 24 NaHCO_3_, 5 KCl, 1.2 KH_2_PO_4_, 2.4 CaCl_2_, 1.3 MgSO_4_, and 10 glucose, aerated with 95% O_2_/5% CO_2_ (to a final pH of 7.4). The standard external solution contained (in mM): 150 NaCl, 5 KCl, 1 MgCl_2_, 2 CaCl_2_, and 10 glucose, buffered to various pH values with either 10 mM HEPES (pH 6.0–7.4), or 10 mM MES (pH<6.0). The standard patch pipette solution for whole-cell patch recording was (in mM): 120 KCl, 30 NaCl, 1 MgCl_2_, 0.5 CaCl_2_, 5 EGTA, 2 Mg-ATP, 10 HEPES. The internal solution was adjusted to pH 7.2 with Tris-base. Unless otherwise noted, the electrophysiological recordings were carried out under standard conditions.

For measurement of the relative permeability of Li^+^, K^+^, and Na^+^, the internal solution contained (in mM): 150 NaCl, 10 EGTA, and 10 HEPES, and the external solution contained (in mM): 150 test monovalent cation (X), 10 HEPES (replaced with MES when pH is 5.0), 10 glucose, and 2 CaCl_2_. The relative permeability of Li^+^ and K^+^ over Na^+^ was measured by comparing the reversal potentials when the external solution contained LiCl, KCl, or NaCl with internal NaCl in each case. The osmolarities of all these solutions were maintained at 300–325 mOsm (Advanced Instruments).

Solutions with different pH values were applied using a rapid application technique termed the “Y-tube” method throughout the experiments [38]. This system allows a complete exchange of external solution surrounding a neuron within 20 ms.

### Site-Directed Mutagenesis

The human ASIC1a cDNA was subcloned into the pEGFPC3 vector (Promega Corporation). Each mutant was generated with the QuikChange mutagenesis kit (Stratagene) in accordance with the manufacturer's protocol.

### Electrophysiological Experiments

The electrophysiological recordings were performed in the conventional whole-cell patch recording configuration under voltage clamp conditions. Patch pipettes were pulled from glass capillaries with an outer diameter of 1.5 mm on a two-stage puller (PP-830, Narishige Co., Ltd.). The resistance between the recording electrode filled with pipette solution and the reference electrode was 4–6 MΩ. Membrane currents or potentials were measured using a patch clamp amplifier (Axon 700A, Axon Instruments) and were sampled and analyzed using a Digidata 1320A interface and a computer with the Clampex and Clampfit software (version 9.0.1, Axon Instruments). In most experiments, 70%–90% series resistance was compensated. Unless otherwise noted, the membrane potential was held at −60 mV throughout the experiment under voltage clamp conditions. All the experiments were carried out at room temperature (22–25°C).

### Data Analysis

Results were expressed as the mean±SEM. Statistical comparisons were made with the Student's *t*-test. The permeability ratios of *p*_Na_/*p*_Li_ and *p*_Na_/*p*_K_ were determined by the modified Goldmann-Holdgkin-Katz equation: *p*_X_/*p*_Na_ = exp (Δ*V*_rev_ *F*/*RT*) due to the equimolar cations in the external and internal solution, where *X* represents the test cation, Δ*V*_rev_ is the change in reversal potential when Na^+^ was replaced by the tested cation, *F* is the Faraday constant, *R* is the gas constant, and *T* is the absolute temperature.

## Supporting Information

Figure S1 **The motions correlation map of residues in subunit A.** Correlated motion maps are represented with a color code related to the sign and intensity of correlations (ranging from dark blue for noncorrelations to dark red for perfect correlations). The color bar on the top indicates the regions of the TM, palm, thumb, and finger domains.
(4.35 MB TIF)

Figure S2 **Bending and swing vibrations between finger and knuckle detected by NMA.** The motions in modes 8, 12, and 16 are shown as examples. The vector arrows represent the amplitude and direction of the displacement experienced by each residue of these two subdomains. The arrows clearly illustrate the bending and swing vibration between them.
(4.30 MB TIF)

Figure S3 **Representative traces and magnitudes of the inward currents for ASIC1 mutants.** (A) Representative traces of the inward currents from CHO cells transfected with WT or mutated ASIC1. (B) Collected data exemplified in (A).
(10.47 MB TIF)

Figure S4 **Typical examples of** ***I/V*** **relationship curve under different conditions.** Channels were activated with pH 5.0 in the whole cells recording. Once currents were fully activated, a fast (800 ms) voltage ramp was run from −90 mV to +90 mV and reverse potential was determined. The application pipette contained (in mM): either 150 NaCl (Na_out_), 150 LiCl (Li_out_), or 150 KCl (K_out_) and 10 glucose, 10 HEPES, and 2 CaCl_2_. The patch pipette contained (in mM): 150 NaCl, 10 HEPES, and 5 EGTA (Na_in_).
(8.66 MB TIF)

Table S1 **Reversal potentials obtained from CHO cells transfected with WT or mutated ASIC1 channel under different conditions.**
(0.04 MB DOC)

Table S2 **Calculated binding free energy (Δ** ***G*** **_binding_) between the thumb and finger subdomains of the WT channel and its mutants.**
(0.03 MB DOC)

Text S1 **Supplemental experimental procedure.**
(0.04 MB DOC)

Video S1 **The shearing vibration of the M-plane in mode 3 induces TM1 and TM2 helices to undergo twisting movements.**
(0.45 MB AVI)

Video S2 **The bending of the M-plane in mode 11 triggers a swinging motion to the TM domain.**
(0.45 MB AVI)

Video S3 **Bending and swing vibrations between finger and knuckle evoke motions of the TM domain through thumb and M-plane in mode 8.** The subunit is shown in ribbon representation with subdomains colored in green (thumb), magenta (finger), cyan (palm), orange (knuckle), blue (β turn), and red (TM).
(0.47 MB AVI)

Video S4 **Bending and swing vibrations between finger and knuckle evoke motions of the TM domain through thumb and M-plane in mode 12.** The subunit is shown as Video S3.
(0.49 MB AVI)

Video S5 **Bending and swing vibrations between finger and knuckle evoke motions of the TM domain through thumb and M-plane in mode 16.** The subunit is shown as Video S3.
(0.48 MB AVI)

## Abbreviations

ASIC: acid-sensing ion channel
EC: extracellular
ENaC: epithelial sodium channel
M-plane: metacarpal plane
MD: molecular dynamics
NMA: normal mode analysis
RMSF: root-mean-square-fluctuation
TM: transmembrane
WT: wild type
