# Supplementary figures and images for: Inherent Dynamics of the Acid-Sensing Ion Channel 1 Correlates with the Gating Mechanism
Source: PLoS Biol. 2009 Jul 14;7(7):e1000151. doi: 10.1371/journal.pbio.1000151 (PMC2701601; doi:10.1371/journal.pbio.1000151)

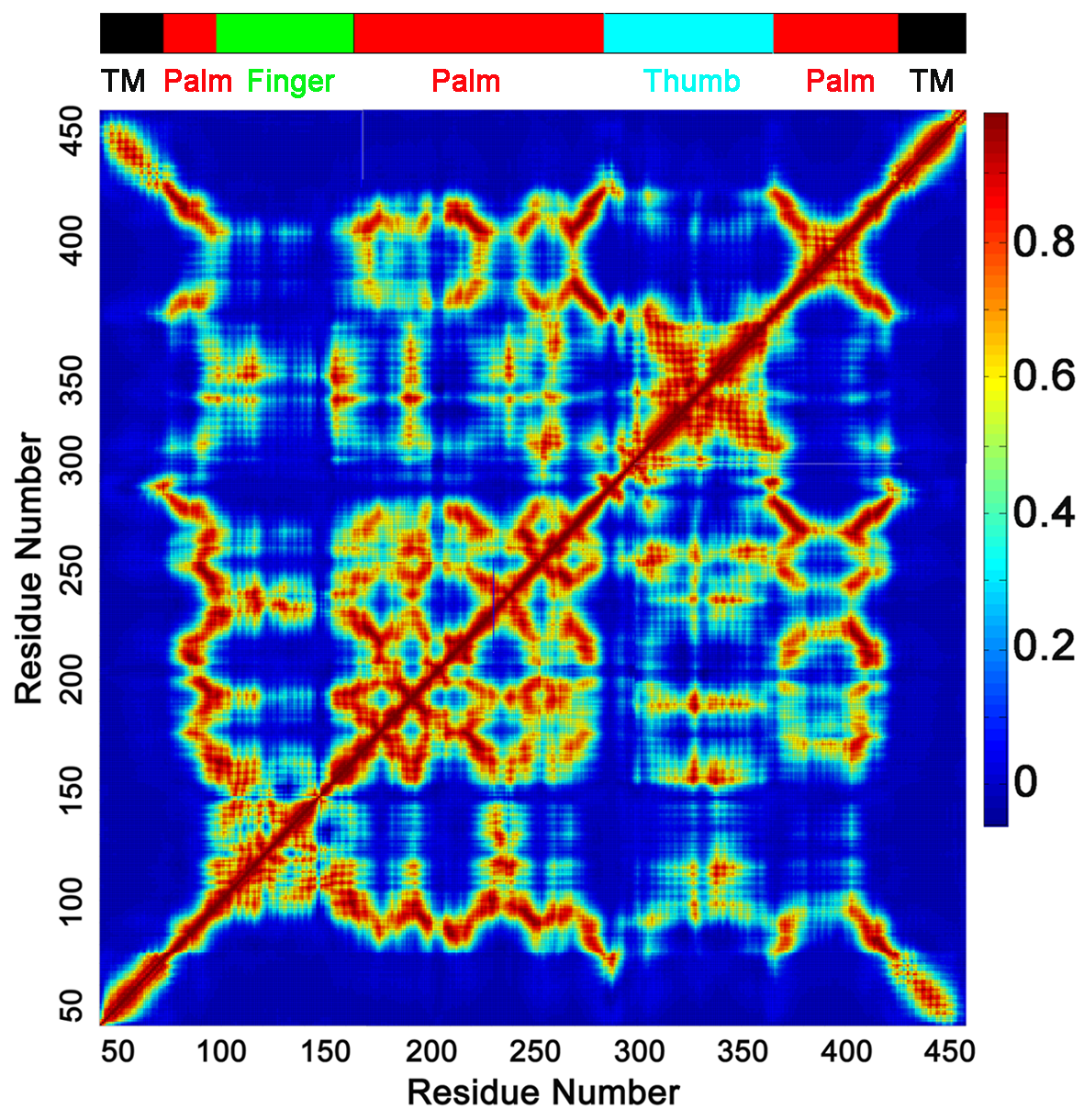

Supplement: Figure S1 — The motions correlation map of residues in subunit A. Correlated motion maps are represented with a color code related to the sign and intensity of correlations (ranging from dark blue for noncorrelations to dark red for perfect correlations). The color bar on the top indicates the regions of the TM, palm, thumb, and finger domains. (4.35 MB TIF) [file pbio.1000151.s001.tif]

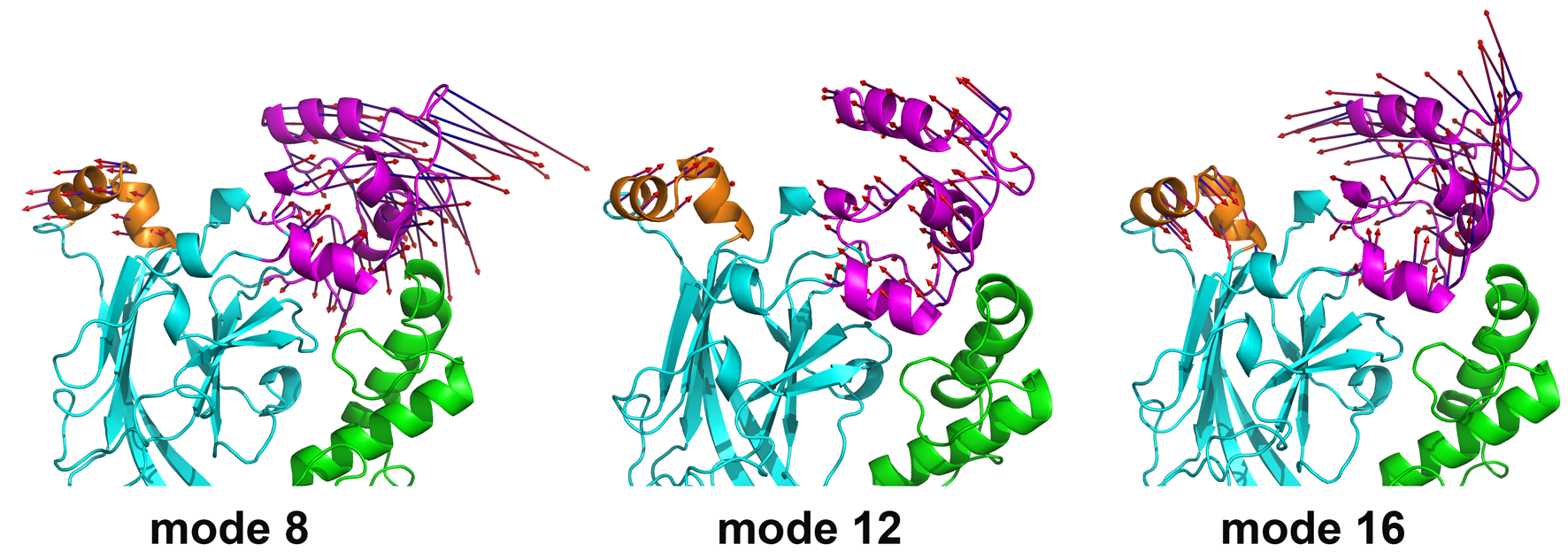

Supplement: Figure S2 — Bending and swing vibrations between finger and knuckle detected by NMA. The motions in modes 8, 12, and 16 are shown as examples. The vector arrows represent the amplitude and direction of the displacement experienced by each residue of these two subdomains. The arrows clearly illustrate the bending and swing vibration between them. (4.30 MB TIF) [file pbio.1000151.s002.tif]

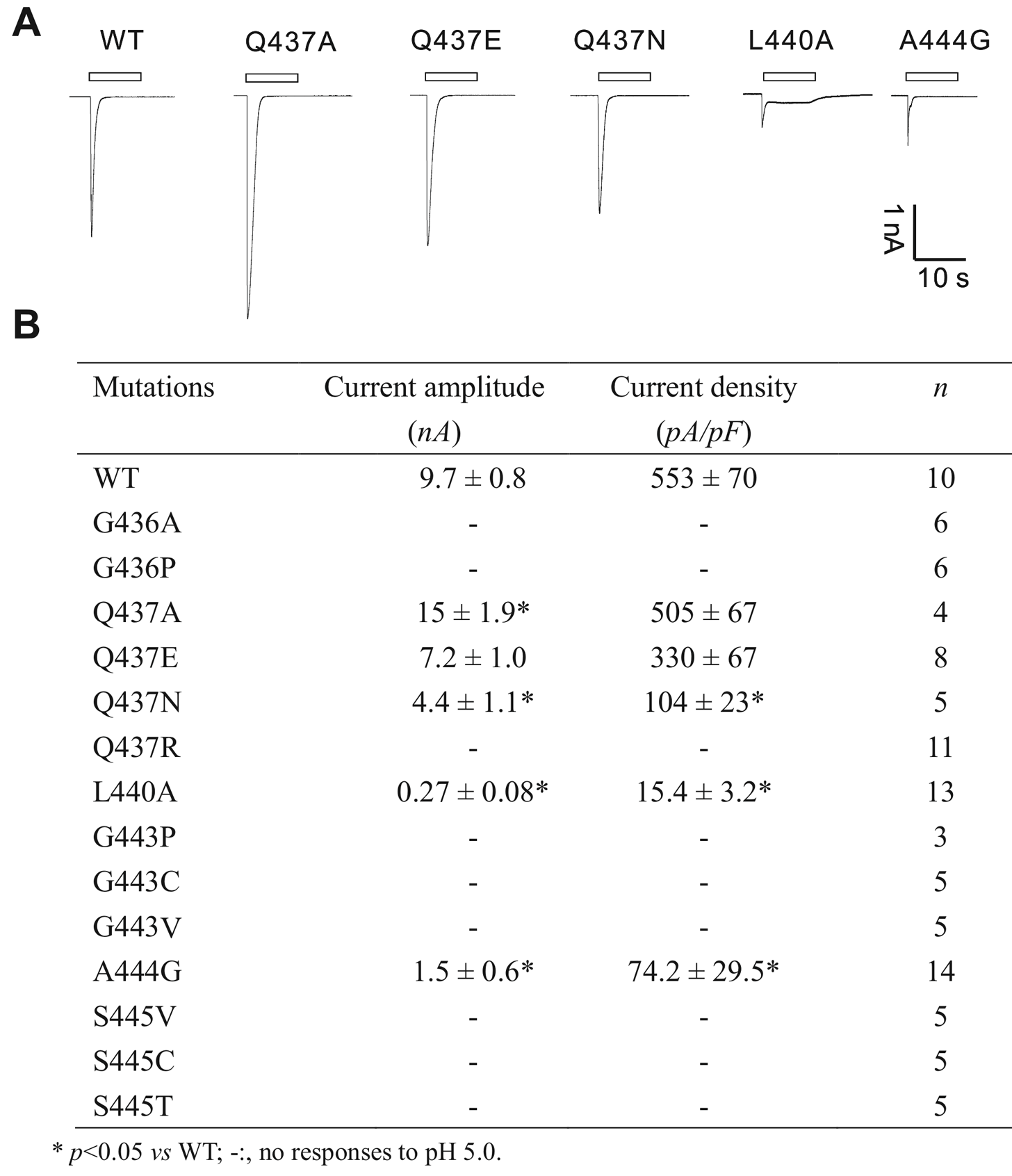

Supplement: Figure S3 — Representative traces and magnitudes of the inward currents for ASIC1 mutants. (A) Representative traces of the inward currents from CHO cells transfected with WT or mutated ASIC1. (B) Collected data exemplified in (A). (10.47 MB TIF) [file pbio.1000151.s003.tif]

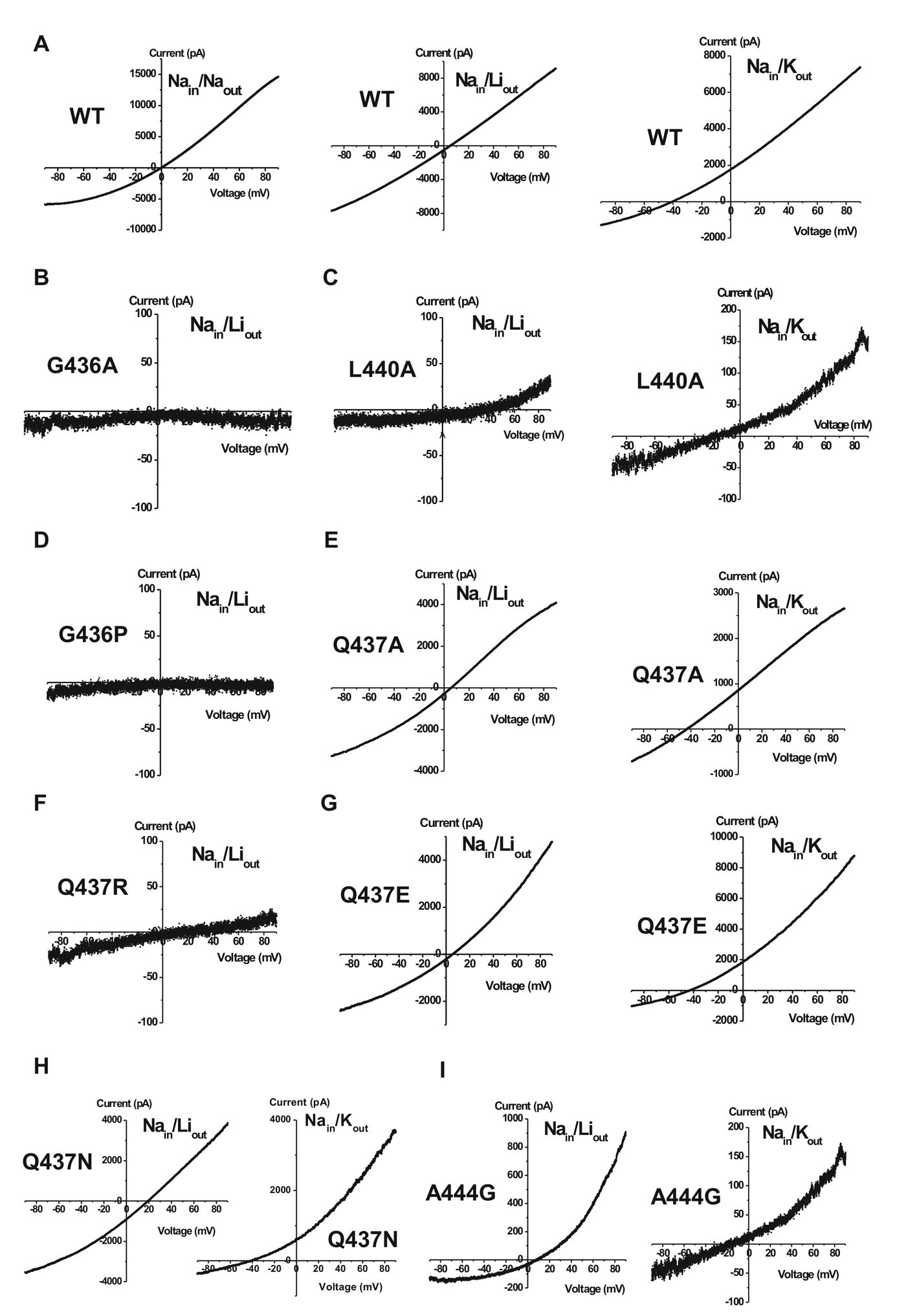

Supplement: Figure S4 — Typical examples of I/V relationship curve under different conditions. Channels were activated with pH 5.0 in the whole cells recording. Once currents were fully activated, a fast (800 ms) voltage ramp was run from −90 mV to +90 mV and reverse potential was determined. The application pipette contained (in mM): either 150 NaCl (Naout), 150 LiCl (Liout), or 150 KCl (Kout) and 10 glucose, 10 HEPES, and 2 CaCl2. The patch pipette contained (in mM): 150 NaCl, 10 HEPES, and 5 EGTA (Nain). (8.66 MB TIF) [file pbio.1000151.s004.tif]
