# Supplementary material for: Inherent Dynamics of the Acid-Sensing Ion Channel 1 Correlates with the Gating Mechanism
Source: PLoS Biol. 2009 Jul 14;7(7):e1000151. doi: 10.1371/journal.pbio.1000151 (PMC2701601; doi:10.1371/journal.pbio.1000151)
Supplement: Table S1 — Reversal potentials obtained from CHO cells transfected with WT or mutated ASIC1 channel under different conditions. (0.04 MB DOC) [file pbio.1000151.s005.doc]

**Table S1.** **Reversal potentials obtained from CHO cells expressing WT or mutated ASIC1 channels under different conditions**

|  | Standard ^a^ | n | Na in /  Li out ^b^ | n | Na in /  K out ^c^ | n |
| --- | --- | --- | --- | --- | --- | --- |
| WT | 38.1 ± 1.1 | 6 | 7.7 ± 0.7 | 12 | -40.2 ± 0.9 | 8 |
| G436A  G436P  Q437A  Q437E  Q437N  Q437R  L440A  G443P  G443C  G443V  A444G | -  -  39.2 ± 3.2  37.8 ± 2.8  41.8 ± 1.1  -  2.6 ± 0.9*  -  -  -  7.3 ± 1.5* | 4  4  5  6  2  4  7  3  5  5  7 | -  -  6.2 ± 0.9  6.9 ± 2.4  15.1 ± 0.9*  -  -  ND  ND  ND  6.8 ± 0.5 | 2  2  16  8  15  3  13  ND  ND  ND  13 | ND  ND  -37.2 ± 1.4  -39.3 ± 4.1  -38.2 ± 2.8  ND  1.3 ± 0.7*  ND  ND  ND  -15.7 ± 1.9* | ND  ND  11  6  9  4  6  ND  ND  ND  ND |
| S445V  S445C  S445T | -  -  - | 5  5  5 | ND  ND  ND | ND  ND  ND | ND  ND  ND | ND  ND  ND |

* *p*<0.05 *vs* WT. - : no responses to pH 5.0; ND: not determined.

^a^ Reversal potential (RP) was obtained by voltage step by step protocol (from -90 to +60 mV) in standard external and internal solutions (see Materials and Methods) from CHO cells transfected with wild-type (WT) or mutated ASIC1.

^b^ RP was obtained by voltage double-ramp protocol (from -90 to +90 mV) in Li^+^-rich external solution (containing 150 mM LiCl, 10 mM glucose, 10 mM HEPES and 2 mM CaCl_2_) and Na^+^-rich internal solution (containing 150 mM NaCl, 10 mM HEPES and 5 mM EGTA) from CHO cells transfected with WT or mutated ASIC1.

^c^ RP was obtained by voltage double-ramp protocol (from -90 to +90 mV) in K^+^-rich external solution (containing 150 mM KCl, 10 mM glucose, 10 mM HEPES and 2 mM CaCl_2_) and Na^+^-rich internal solution (containing 150 mM NaCl, 10 mM HEPES and 5 mM EGTA) from CHO cells transfected with WT or mutated ASIC1.
