# Supplementary material for: Inherent Dynamics of the Acid-Sensing Ion Channel 1 Correlates with the Gating Mechanism
Source: PLoS Biol. 2009 Jul 14;7(7):e1000151. doi: 10.1371/journal.pbio.1000151 (PMC2701601; doi:10.1371/journal.pbio.1000151)
Supplement: Table S2 — Calculated binding free energy (Δ G binding) between the thumb and finger subdomains of the WT channel and its mutants. (0.03 MB DOC) [file pbio.1000151.s006.doc]

**Table S2. Calculated Binding Free Energy (Δ*G*_binding_) Between the Thumb and Finger Sub-domains of the Wild-type (WT) Channel and Its Mutants.**

|  | Δ*G*_binding_ (kcal/mol) |
| --- | --- |
| WT | -58.72 ± 7.20 |
| R191A | -30.33 ± 7.64 |
| R191E | -33.88 ± 7.90 |
| D238A | -46.50± 6.93 |
| D238K | -69.26 ± 6.60 |
| D238N | -51.05 ± 8.60 |
| D238S | -61.76± 8.86 |
| E239K | -46.49 ± 8.90 |
| E239Q | -55.14 ± 7.09 |
| H328A | -41.54 ± 10.42 |
| P338A | -49.99 ± 6.18 |
| D346N | -43.27±9.57 |
