# Supplementary material for: Inherent Dynamics of the Acid-Sensing Ion Channel 1 Correlates with the Gating Mechanism
Source: PLoS Biol. 2009 Jul 14;7(7):e1000151. doi: 10.1371/journal.pbio.1000151 (PMC2701601; doi:10.1371/journal.pbio.1000151)
Supplement: Text S1 — Supplemental experimental procedure. (0.04 MB DOC) [file pbio.1000151.s007.doc]

**Supplemental Experimental Procedure**

**Binding Free Energy Calculation**

The initial structural models for calculating the binding free energies between the thumb and finger for the wild-type (WT) channel and its mutants were constructed based on the X-ray crystal structure of cASIC1 (Protein Data Bank entry 2QTS) (Jasti et al., 2007). For the WT complex, residues 71–290 and 367–424 were used for the thumb and residues 292–365 were used for the finger. For each finger-thumb complex mutant, the corresponding residue in the WT complex was mutated by the appropriate residue using the Insight II program (Accelrys, San Diego, CA). In this study, structural models of seven mutant complexes were constructed: Arg191Ala, Arg191Glu, Asp238Lys, Glu239Lys, Glu239Gln, His238Ala and Pro338Ala. The complex was solvated into a box of SPC (Simple Point Charge) water molecules (Berendsen et al., 1981) with a minimal distance of 10 Å between the box boundary to any protein atom. The protein/water system was submitted to energy minimization. An appropriate number of ions were added to neutralize the system. The system was subsequently minimized again. The system was then subjected to a 700-ps molecular dynamics (MD) simulation.

MD simulations were carried out by using the AMBER 9.0 package (Case et al., 2006) with constant temperature, constant pressure (NPT), and periodic boundary conditions. The Amber Parm03 force field (Duan et al., 2003) was applied for the proteins. The Particle Mesh Ewald (PME) method (Darden et al., 1993; Essmann et al., 1995) was used to calculate the long-range electrostatics interactions. The nonbonded cutoff was set to 8.0 Å, and the nonbonded pairs were updated every 25 steps. The SHAKE method (Ryckaert et al., 1977) was applied to constrain all covalent bonds involving hydrogen atoms. Each simulation was coupled to a 300 K thermal bath at 1.0 atm pressure (Berendsen et al., 1984). The temperature and pressure coupling parameters were set as 0.2 and 0.05 ps, respectively. An integration step of 2 fs was used for the MD simulations.

After the 700-ps MD simulations, the binding free energy (ΔG_binding_) between thumb and finger of each complex was calculated by using the MM-PBSA method encoded in the AMBER 9.0 program (Case et al., 2006). Snapshots without the water molecules extracted from the MD trajectories between 400–700 ps were used in the MM-PBSA calculations. For each snapshot, the thumb-finger binding free energy (Δ*G*_binding_) was calculated using equation 4 (Srinivasan et al., 1998),

Δ*G*_binding_ = *G*_complex_ – [*G*_thumb_ + *G*_finger_] (4)

where *G*_complex_, *G*_thumb_ and *G*_finger_ are the free energies of the complex, thumb and finger, respectively. Each free energy term in equation (4) was calculated with the absolute free energy of the species (thumb, finger and their complex) in gas phase (*E*_gas_), the solvation free energy (*G*_solvation_) and the entropy term (*TS*) using equation (5):

*G* = *E*_gas_ + *G*_solvation_ – *TS*  (5)

*E*_gas_ is the sum of the internal strain energy (*E*_int_), van der Waals energy (*E*_vdW_) and electrostatic energy (*E*_ele_) (equation (6)). *E*_int_ is the energy associated with vibrations of covalent bonds and bond angles and the rotation of single bond torsional angles (equation (7)).

*E*_gas_ = *E*_int_ + *E*_vdW_ + *E*_ele_ (6)

*E*_int_ = *E*_bond_ + *E*_angle_ + *E*_torsion_ (7)

The solvation free energy, *G_s_*_olvation_, is approximated as the sum of the polar contribution (*G*_PB_) and nonpolar contribution (*G*_nonpolar_) using a continuum representation of the solvent:

*G*_solvation_ = *G*_PB_ + *G*_nonpolar_ (8)

*G*_nonpolar_ = γ × SASA + *b* (9)

The polar contribution (*G*_PB_) to the solvation energy was calculated using the DELPHI program with PARSE (Honig and Nicholls, 1995) atom radii and standard Parm03 (Duan et al., 2003) charges for amino acids implemented in AMBER 9.0. The grid size used was 0.5 Å. The dielectric constant was set to 1 for interior solute and 80 for exterior water. Each PB calculation was run to convergence (the maximum change in potential was less than 0.001 *kT*/*e*). The nonpolar contributions (*G*_nonpolar_) were estimated using equation (9) (Sitkoff et al., 1994), where SASA is the solvent-accessible surface area that was estimated using the MSMS algorithm with a probe radius of 1.4 Å (Sanner et al., 1996). The surface tension proportionality constant γ and the free energy of nonpolar solvation for a point solute *b* were set to 0.00542 kcal/mol•Å^-2^ and 0.92 kcal/mol, respectively, which were widely used in the simulations of other systems (Kuhn et al., 2005; Massova and Kollman, 1999; Moreira et al., 2006; Swanson et al. 2004; Wang et al., 2001). To reduce computational time, the entropic contribution (*TS*) to the binding free energy was not calculated for the systems in this study, because the entropic contributions are expected to be canceled when the relative binding free energies are calculated between the wild-type and mutants.

**Supplemental References**

Berendsen, H.J.C., Postma, J.P.M., van Gunsteren, W.F., and Haak., J.R. (1984). Molecular dynamics with coupling to an external bath. J. Chem. Phys. *81*, 3684–3690.

Berendsen, H.J.C., Postma, J.P.M., van Gunsteren, W.F., and Hermans, J. (1981). Interaction models for water in relation to protein hydration. In Intermolecular Force. B. Pullman, ed. (Dordrecht, The Netherlands: D. Reidel Publishing Company), pp. 331–342.

Case, D.A., Darden, T., Cheatham, T.E., 3rd, Simmerling, C., Wang, J., Duke, R.E., Luo, R., Merz, K.M., Pearlman, D.A., Crowley, M., et al. AMBER 9. (2006) San Francisco, CA: University of California.

Darden, T.A., York, D.M., and Pedersen, L.G. (1993). Particle mesh Ewald: An N·log(N) method for Ewald sums in large systems. J. Chem. Phys. *98*, 10089–10092.

Duan, Y., Wu, A., Chowdhury, C.S., Lee, M.C., Xiong, G., Zhang, W., Yang, R., Cieplak, P., Luo, R., Lee, T., Caldwell, J., Wang, J., and Kollman, P. (2003). A point-charge force field for molecular mechanics simulations of proteins based on condensed-phase quantum mechanical calculations. J. Comput. Chem. *24*, 1999–2012.

Essmann, U., Perera, L., Berkowitz, M.L., Darden, T., Lee, H., and Pedersen, L.G. (1995). A smooth particle mesh Ewald method. J. Chem. Phys. *103*, 8577–8592.

Honig, B., and Nicholls, A. (1995). Classical electrostatics in biology and chemistry. Science *268*, 1144–1149.

Jasti, J., Furukawa, H., Gonzales, E.B., and Gouaux, E. (2007). Structure of acid-sensing ion channel 1 at 1.9Ǻ resolution and low pH. Nature *449*, 316–323.

Kuhn, B., Gerber, P., Schulz-Gasch, T., and Stahl, M. (2005). Validation and use of the MM-PBSA approach for drug discovery. J. Med. Chem. *48*, 4040–4048.

Massova, I., and Kollman, P.A. (1999). Computational alanine scanning to probe protein-protein interactions: a novel approach to evaluate binding free energies. J. Am. Chem. Soc. *121*, 8133–8143.

Moreira, I.S., Fernandes, P.A., and Ramos, M.J. (2006). Unraveling the importance of protein-protein interaction: application of a computational alanine-scanning mutagenesis to the study of the IgG1 streptococcal protein G (C2 fragment) complex. J. Phys. Chem. B *110*, 10962–10969.

Ryckaert, J.P., Ciccotti, G. and Berendsen, J.C. (1977). Numerical integration of the cartesian equations of motion of a system with constrains: molecular dynamics of n-alkanes J. Comput. Phys. *23*, 327–341.

Sanner, M.F., Olson, A.J., and Spehner, J.C. (1996). Reduced surface: an efficient way to compute molecular surfaces. Biopolymers *38*, 305–320.

Sitkoff, D., Sharp, K.A., and Honig, B. (1994). Accurate Calculation of Hydration Free Energies Using Macroscopic Solvent Models. J. Phys. Chem. *98*, 1978–1988.

Srinivasan, J., Miller, J., Kollman, P.A., and Case, D.A. (1998). Continuum solvent studies of the stability of RNA hairpin loops and helices. *J. Biomol. Struct. Dyn.*, *16*, 671–682.

Swanson, J.M.J., Henchman, R.H., and McCammon, J.A. (2004). Revisiting free energy calculations: a theoretical connection to MM/PBSA and direct calculation of the association free energy. Biophys. J. *86*, 67–74.

Wang, J.M., Morin, P., Wang, W., Kollman, P.A. (2001). Use of MM-PBSA in reproducing the binding free energies to HIV-1 RT of TIBO derivatives and predicting the binding mode to HIV-1 RT of efavirenz by docking and MM-PBSA. J. Am. Chem. Soc. *123*, 5221–5230.
